# Identification of a novel *NKX2-5* variant in a young Ecuadorian patient with atrioventricular block and bradycardia: a case report

**DOI:** 10.3389/fcvm.2025.1552423

**Published:** 2025-04-04

**Authors:** Viviana A. Ruiz-Pozo, Santiago Cadena-Ullauri, Elius Paz-Cruz, Rafael Tamayo-Trujillo, Patricia Guevara-Ramirez, Paul Onofre-Ruiz, Ana Karina Zambrano

**Affiliations:** ^1^ Universidad UTE, Facultad de Ciencias de la Salud Eugenio Espejo, Centro de Investigación Genética y Genómica, Quito, Ecuador; ^2^ Universidad UTE, Facultad de Ciencias de la Salud Eugenio Espejo, Quito, Ecuador

**Keywords:** case report, cardiovascular disease, genetics, genomics, healthcare

## Abstract

Cardiovascular diseases (CVDs) are the leading global cause of mortality, with South America reflecting similar trends. Among congenital heart diseases (CHDs), atrioventricular (AV) block is included. AV block is a condition defined by abnormal electrical signal transmission between the atria and ventricles. Advances in Next-Generation Sequencing (NGS) have facilitated the identification of genetic variants associated with cardiac disorders, such as AV block. Notably, the transcription factor NKX2-5 plays a crucial role in heart development and function, and mutations in this gene have been linked to bradycardia and AV block. This article describes the case report of a young Ecuadorian child diagnosed with AV block and bradycardia. Furthermore, by performing NGS, a missense variant, p.(Tyr274Ser) substitution, in the *NKX2-5* gene has been identified and classified as a variant of uncertain significance (VUS). Ancestral analysis has shown a genetic background of 16.5% African, 45.9% European, and 37.6% Native American. These findings suggest a potential association between the identified *NKX2-5* variant and the patient's phenotype, highlighting the importance of integrating genomic and ancestral analyses to advance personalized diagnostics and therapeutics in diverse populations, such as the mestizo population.

## Introduction

Cardiovascular diseases (CVDs) are the leading cause of mortality worldwide, and the situation in South America mirrors this trend (1, 2). CVDs encompass a range of disorders affecting the heart and blood vessels, including congenital heart diseases (CHD), which affect the functioning and development of cardiac structures (3). Among CHDs, atrioventricular (AV) block is one of the most common disorders (4), characterized by impaired transmission of electrical signals from the atria to the ventricles (5). A common sign of AV block is bradycardia, defined as a heart rate below the age-specific lowest normal threshold (6, 7).

Genomic screenings are fundamental in diagnosing CVDs and developing personalized treatment strategies (1, 8–10). In this context, Next-Generation Sequencing (NGS) has been established as a valuable tool for identifying genetic variants associated to various cardiac conditions (11–13). Notably, numerous genes have been implicated in bradycardia and AV block (7), including the *NKX2-5* gene, a key transcription factor involved in normal heart development and function (14, 15).

Ethnicity and ancestry have also been linked to the prevalence of cardiac conditions (16). For instance, Black adults exhibit the highest prevalence of hypertension, a condition strongly associated with multiple cardiac diseases (17).

This report describes a novel *NKX2-5* variant in an Ecuadorian child with AV block and bradycardia.

## Case presentation

In June 2019, a 6-month-old male infant was referred to a cardiologist for evaluation. Upon examination, an arrhythmia associated with bradycardia was detected in the absence of other signs or symptoms. Based on these findings, an electrocardiogram (ECG) was requested for further assessment.

**Figure 1:**
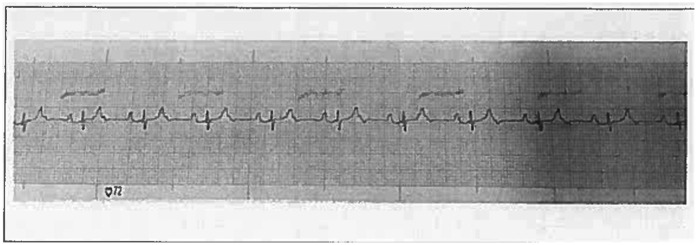
Electrocardiogram of the individual at 6 months of age.

The ECG revealed a heart rate of 68 beats per minute (bpm) with a narrow QRS complex measuring 60 ms in duration. An R wave in V1 and an S wave in V6 were observed, without electrocardiographic evidence of significant right ventricular enlargement. The PR interval was prolonged at 24 ms, indicating the presence of a 2:1 AV block (Figure 1).

**Figure 2:**
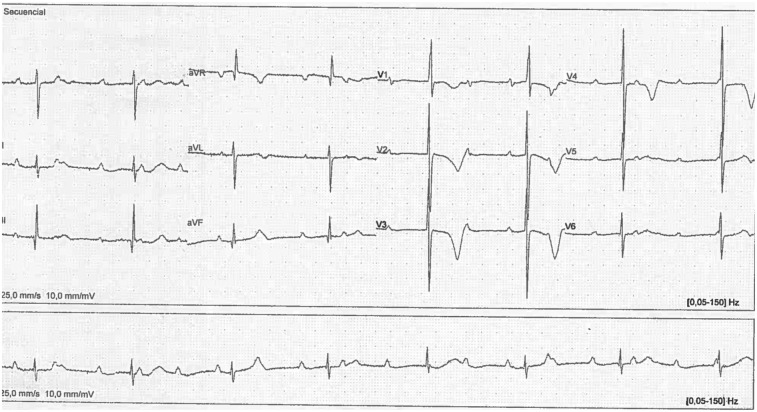
Electrocardiogram of the individual at 22 months of age.

An echocardiogram was performed, revealing no apparent structural or functional abnormalities. The patient exhibited situs solitus with AV concordance. Both atria were of normal size, with an intact interatrial septum. The tricuspid valve demonstrated normal morphology and function, and the right ventricle was of normal size with preserved systolic performance. The interventricular septum was classified as Type I. The left ventricle also exhibited normal size and function, with both the left and right ventricular outflow tracts appearing structurally and functionally normal. The aortic and pulmonary valves, as well as the pulmonary trunk and its branches, were morphologically and functionally normal.

In September 2019, a 20-h and 30-min Holter monitoring was performed. The report indicated a mean heart rate of 70 bpm, with a maximum peak of 91 bpm. No ST segment abnormalities were detected, and no significant heart rate variations were observed compared to the resting electrocardiogram. Consequently, periodic medical follow-up was recommended.

In October 2020, at 1 year and 10 months of age, the patient was evaluated by a specialist for follow-up. The medical history was negative for syncopal episodes; however, brief episodes of perioral cyanosis during crying were reported. Psychomotor development was appropriate, and growth parameters were within normal limits. Cardiac examination revealed a grade II holosystolic murmur, an accentuated second heart sound (R2), and a hyperdynamic precordium. Based on these findings the specialist requested another electrocardiogram and echocardiogram.

The electrocardiogram revealed a heart rate of 46 beats per minute (bpm), a narrow QRS complex, an RV1 pattern and an SV6 wave indicative of right ventricular enlargement. In addition, a variable PR interval, right axis deviation and evidence of complete AV block (third-degree) were observed (Figure 2).

The echocardiogram performed during this evaluation revealed mild tricuspid regurgitation, dilation of both the right and left ventricles, and an estimated pulmonary pressure of 55 mmHg, confirming the diagnosis of moderate to severe pulmonary hypertension (Table 1). Given that the patient remained asymptomatic with preserved ventricular function, conservative management with enalapril was initiated.

**Table 1:** Echocardiographic findings from 2019 to 2020.

Parameters	Echocardiogram (2019)	Echocardiogram (2020)
RVD dimensions	1.7 cm	1.8
TAPSE	1.9 mm	2.2 mm
Pulmonary flow Doppler	2.76 mmHg	1.9 mmHg
Ejection fraction (EF)	73	78
LVEDD	1.5	1.9
Tricuspid insufficiency	0 mmHg	68 mmHg

**Figure 3:**
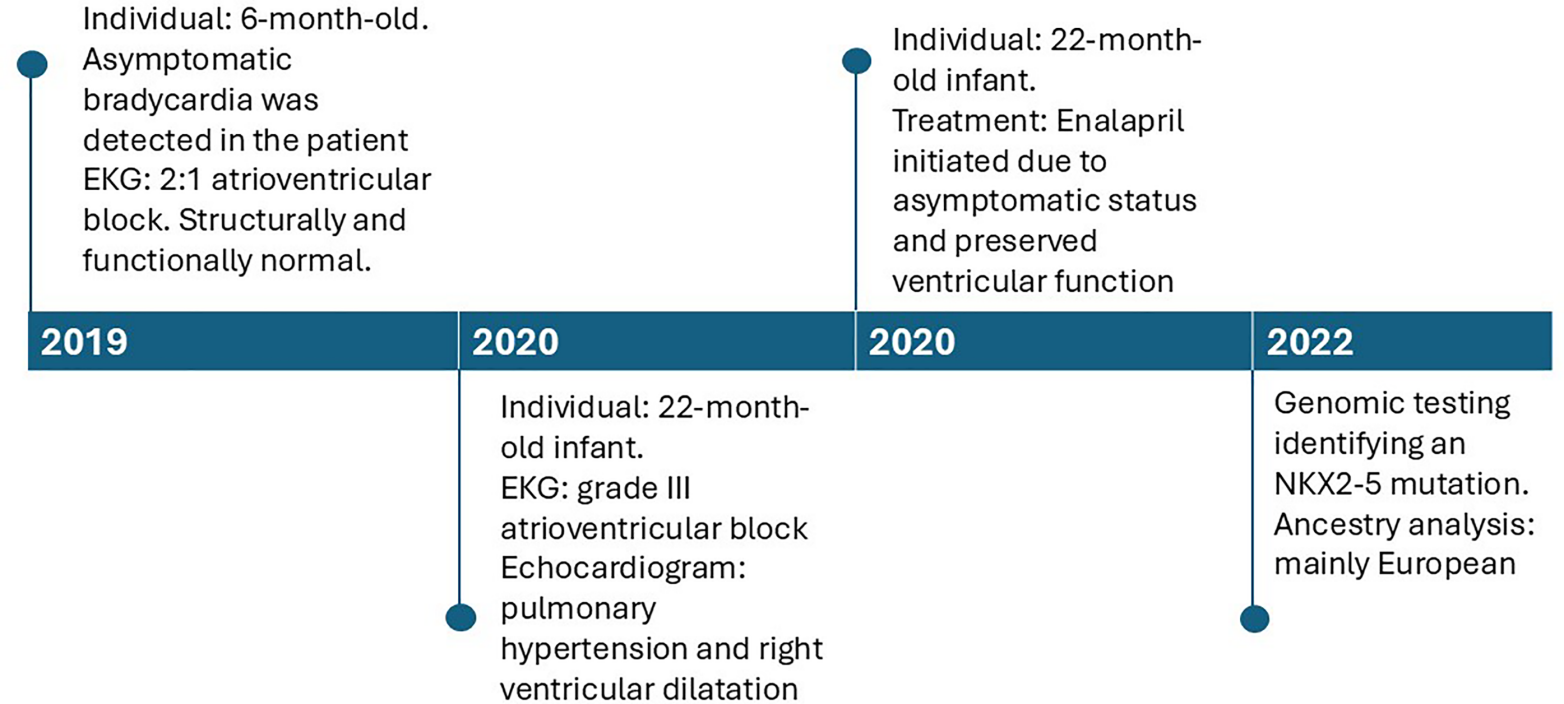
Timeline depicting the key episodes of care.

A timeline of the relevant episodes of care is depicted in Figure 3.

**Figure 4:**
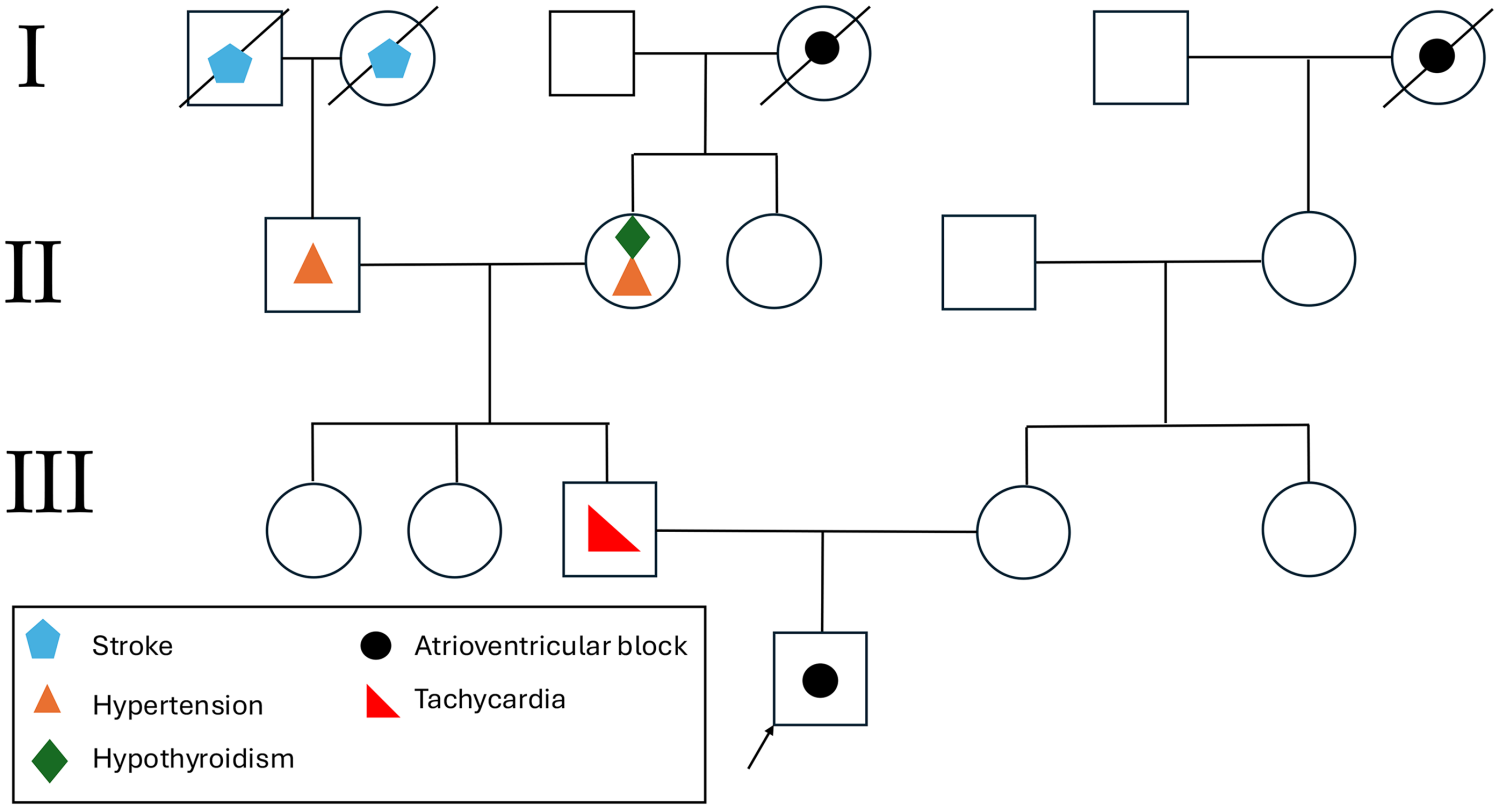
Representation of the proband's pedigree. Affected family members are marked with disease-specific symbols, with a legend in the bottom left explaining the symbols used.

Furthermore, the patient had a significant family history of cardiovascular disease. His father was diagnosed with tachycardia at 18 years of age and underwent catheter ablation. The paternal grandfather had a history of hypertension, and the paternal great-grandmother had a medical history of heart disease and suffered a stroke. A detailed pedigree illustrating the family history is provided in Figure 4.

## Materials and methods

### Ethical considerations

For participation in this study, the legal representative provided written informed consent, and the patient gave informed assent.

#### DNA extraction and next generation sequencing (NGS)

DNA extraction was extracted from peripheral blood using the PureLinkTM Genomic DNA Mini Kit. Quantification and quality assessment were performed using spectrophotometric and fluorometric methods. NGS was performed at the Center for Genetic and Genomic Research (CIGG) following the manufacturer's protocol for the TruSight™ Cardio (TSC) sequencing panel (Illumina MiSeq). The TSC sequencing panel includes 174 genes related to 17 inherited cardiovascular diseases.

Bioinformatics analyses were performed using DRAGEN Enrichment v3.9.5, Annotation Engine v3.15, PolyPhen, SIFT, and Variant Interpreter v2.16.1.300 platforms were used.

#### Ancestry analysis

Ancestry analysis was conducted using 46 ancestry-informative INDEL markers (AIMs) through a multiplex polymerase chain reaction (PCR), following the protocol described by Zambrano et al. (15). Fragment detection was performed using the Genetic Analyzer 3500 (Applied Biosystems, USA) was used to detect fragments. Data collection and analysis were conducted using Gene Mapper v.5 and Data Collection v.3.3. Ancestry composition was determined using the STRUCTURE v.2.3.4 software (18).

## Results

Genomic analysis of the patient was performed using NGS. The results revealed that 98.42% of the target regions in the TSC Sequencing Panel had a coverage of ≥20×. A variant of uncertain significance (VUS) was identified, potentially associated with the patient's cardiac condition. The detected missense variant, c.821A >C, resulted in a p.(Tyr274Ser) substitution in the *NKX2-5* gene.

Moreover, an ancestral composition analysis was performed, and the results revealed 16.5% African, 45.9% European, and 37.6% Native American components.

## Discussion

In the present study, a novel Tyr274Ser mutation in the *NKX2-5* gene was identified in a patient with bradycardia caused by an AV block. The cardiac homeobox protein NKX2-5 is essential for multiple stages of cardiac development. Numerous studies have indicated that mutations in this gene lead to a wide range of congenital cardiac malformations, including AV conduction disorders, and atrial septal defects (ASD), with variable expressivity and penetrance (19, 20).

Several studies have reported that mutations in the *NKX2-5* gene, in addition to potentially leading to ASD-II, could also result to the development of AV block (21–25). Briggs et al. demonstrated that NKX2-5 is crucial for cardiac conduction and contraction by regulating the expression of several ion channel genes, including SCN5A, T-type Ca^2+^ channels (α1G and α1H), and RyR2. These findings suggest that *NKX2-5* is not only involved in atrial septum formation but is also important in AV node development and the regulated expression of ion channels involved in cellular contraction and conduction (26).

Schott et al. analyzed the *NKX2-5* gene in four families with ASD-II and AV block, identifying three distinct heterozygous mutations. Among 33 affected individuals, 27 had ASD, and all those with available clinical data exhibited AV conduction defects, suggesting that some variant carriers may experience conduction disturbances without ASD. Additionally, eight patients had other structural heart defects, including ventricular septal defect (VSD), tetralogy of Fallot (TOF), aortic subvalvular stenosis, left ventricular hypertrophy, pulmonary atresia, and redundant mitral leaflets with fenestrations (27).

Two of the mutations were predicted to impair *NKX2-5* binding to target DNA, resulting in haploinsufficiency, while the third mutation could potentially enhance binding to target DNA. These findings indicate that *NKX2-5* is crucial for the regulation of septation during cardiac morphogenesis and for the maturation and maintenance of AV node function throughout life (27).

The 324-residue NKX2-5 protein has three conserved domains: Tinman (TN), homeobox (HD), and NK2-specific domain (NK2-SD). While numerous *NKX2-5* mutations have been reported across its sequence, this is the first time a missense mutation at residue 274 has been identified (28).

In this study, a well-conserved, aromatic, and hydrophobic tyrosine at position 274 in the NKX2-5 amino acid sequence, was substituted by the neutral, polar amino acid serine (Tyr274Ser). Various computational platforms were used to assess the potential impact of this variant. The Variant Interpreter platform classified Tyr274Ser as a VUS. Additionally, the Combined Annotation-Dependent Depletion (CADD) tool, which predicts the deleteriousness of genomic variants, assigned this variant a score of 22.8, suggesting a “quite likely deleterious” effect (29). In contrast, predictions from AlphaMissense and Evolutionary Scaled Model (ESM-1b) classified the variant as benign (30, 31). Based on these results, this variant has been catalogued as a VUS using the American College of Medical Genetics and Genomics (ACMG) and the Association for Molecular Pathology (AMP) guidelines (32, 33).

To further investigate the conservation of this region, a protein BLAST analysis utilizing the NCBI tool identified the Tyr 274 residue across multiple mammalian species. Moreover, sequence alignments from UniRef90, analyzed with the ScoreCons algorithm, yielded a conservation score of 0.46, classifying this position as “moderate conservation” (34).

Interestingly, the subject in this study does not have ASD-II, but has a progressive AV block that has now advanced to third-degree AV block. Similarly, Morlanes-Gracia et al. described a family carrying an *NKX2-5* mutation in which not all family members exhibited the same phenotype. Instead, individuals presented various cardiac disturbances, including ASD, conduction disorders, and tachyarrhythmias. This underscores the variable penetrance and expressivity of *NKX2-5* mutations and their association with diverse cardiac phenotypes (35). Likewise, Jhaveri et al. presented a case involving two male siblings carrying a *NKX2-5* mutation, concluding that children and young adults with *NKX2-5* mutations may present a broad spectrum of cardiac disturbances, affecting both electrical conduction as well as structural integrity (36).

Although the discovered mutation alters the amino acid sequence of NKX2-5 at position 274, the HD and NK2-SD domains remain intact. This suggests that the observed phenotype is unllikely due to decreased DNA-binding activity, as the homeodomain remains unchanged. Instead, this mutation may exert dominant-negative effects, potentially altering the protein's affinity for transcriptional partners (19). These findings could provide new insights into the underlying genetic mechanisms of AV block and could contribute to the development of novel therapeutic approaches.

Furthermore, Kalayinia et al. conducted a population study to assess *NKX2-5* mutation prevalence. Among 105 identified mutations, the highest prevalence was observed in European populations (24.1%), while the Latin American populations exhibited a lower prevalence (4.1%) (37). Interestingly, despite being Ecuadorian, a country with a high Native American ancestral component, the patient in this case report has a higher proportion of European ancestry, which may be associated with an increased susceptibility to *NKX2-5* mutations.

The limitations in this case report derive from the inherent challenges associated with NGS. These include the requirement for specialized equipment, the necessity of expertise in this technology, and the reliance on advanced bioinformatic tools for data analysis. Furthermore, NGS is relatively more expensive than other technologies, which limits its access particularly in countries like Ecuador (8, 11, 13).

Despite these constraints, NGS offers significant advantages over other available techniques, such as Sanger sequencing. Notable strengths of NGS include its ability to achieve higher genomic coverage, analyze multiple genes simultaneously, and provide results with greater confidence (8).

## Conclusion

This case report describes a novel *NKX2-5* mutation, Tyr274Ser, identified in a patient with progressive AV block. The study contributes to the growing body of evidence on the role of *NKX2-5* variants in cardiac conduction disorders. Notably, this is the first time a missense mutation at residue 274 has been reported, emphasizing the significance of genetic analysis in uncovering new pathogenic variants.

The findings further highlight the variable expressivity and incomplete penetrance associated with *NKX2-5* mutations. While many carriers of NKX2-5 variants present with ASD, the patient in this study exhibited an isolated conduction defect, reinforcing the heterogeneous phenotypic manifestations of these mutations. This underscores the need for comprehensive clinical and genetic evaluations to better characterize genotype-phenotype correlations.

Moreover, ancestry determination played a crucial role in this study, as the patient, despite being from Ecuador showed a higher proportion of European ancestry, which has been linked to an increased prevalence of *NKX2-5* mutations. This observation suggests that population-specific genetic backgrounds may influence the susceptibility to congenital heart diseases, an aspect that warrants further investigation.

## Data Availability

The original contributions presented in the study are publicly available. This data can be found here: https://www.ncbi.nlm.nih.gov/bioproject/PRJNA1238311/, accession number: PRJNA1238311.
